# Supplementary figures and images for: The shift of percent excess mortality from zero-COVID policy to living-with-COVID policy in Singapore, South Korea, Australia, New Zealand and Hong Kong SAR
Source: Front Public Health. 2023 Mar 20;11:1085451. doi: 10.3389/fpubh.2023.1085451 (PMC10067885; doi:10.3389/fpubh.2023.1085451)

S1A. Singapore:

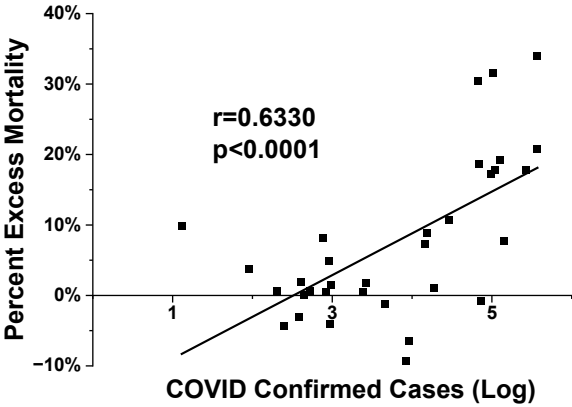

S1B. South Korea:

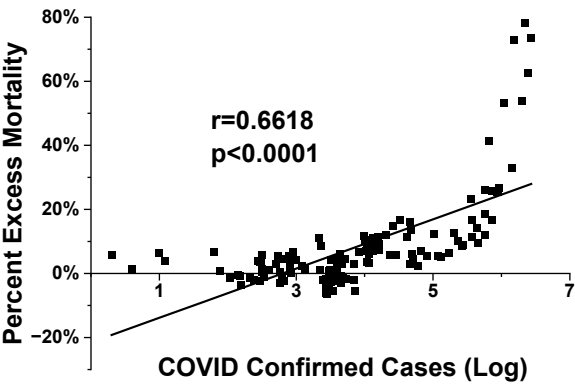

S1C. Australia:

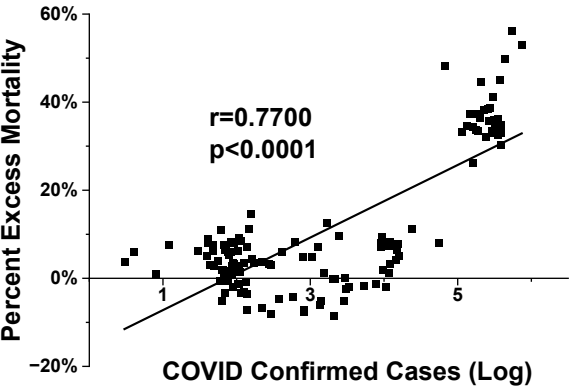

S1D. New Zealand:

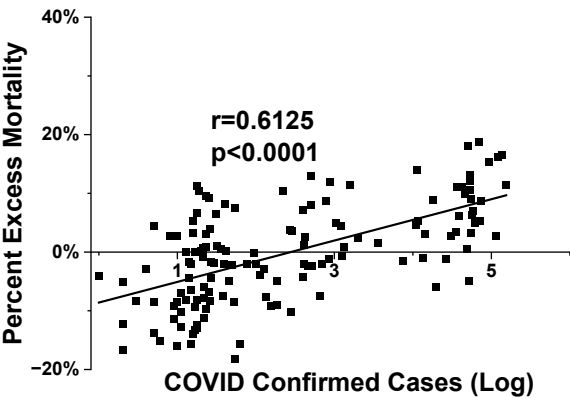

S1E. Hong Kong:

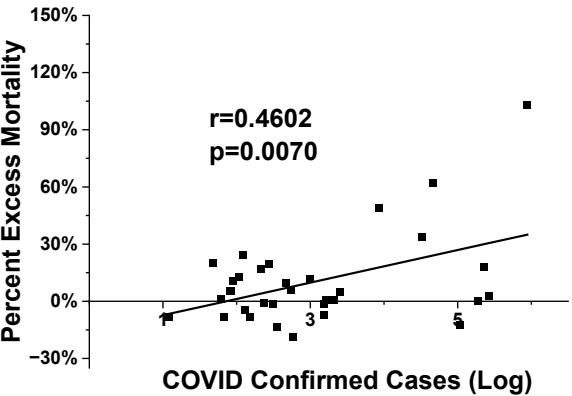

Supplement: Supplementary Figure 1 — Pearson's correlation analysis were performed to compare the correlation between PEM and confirmed COVID cases (Log value) in (A) Singapore, (B) South Korea, (C) Australia, (D) New Zealand and (E) Hong Kong. A p < 0.05 was considered statistically significant. [file Data_Sheet_1.PDF]
